# RMH-YOLO: A Refined Multi-Scale Architecture for Small-Target Detection in UAV Aerial Imagery

**DOI:** 10.3390/s25227088

**Published:** 2025-11-20

**Authors:** Fan Yang, Min He, Jiuxian Liu, Haochen Jin

**Affiliations:** 1 Highway School, Chang’an University, Xi’an 710064, China; 2019021069@chd.edu.cn (F.Y.); ljxjob@chd.edu.cn (J.L.); haochen.jin@chd.edu.cn (H.J.); 2 School of Architecture and Civil Engineering, Xi’an University of Science and Technology, Xi’an 710064, China; 3 Research Center of Highway Large Structure Engineering on Safety, Ministry of Education, Chang’an University, Xi’ an 710064, China

**Keywords:** small-target detection, UAV imagery, dual-attention mechanism, multi-scale fusion, aerial monitoring

## Abstract

Unmanned aerial vehicle (UAV) vision systems have been widely deployed for aerial monitoring applications, yet small-target detection in UAV imagery remains a significant challenge due to minimal pixel representation, substantial scale variations, complex background interference, and varying illumination conditions. Existing object detection algorithms struggle to maintain high accuracy when processing small targets with fewer than 32 × 32 pixels in UAV-captured scenes, particularly in complex environments where target-background confusion is prevalent. To address these limitations, this study proposes RMH-YOLO, a refined multi-scale architecture. The model incorporates four key innovations: a Refined Feature Module (RFM) that fuses channel and spatial attention mechanisms to enhance weak feature representation of small targets while maintaining contextual integrity; a Multi-scale Focus-and-Diffuse (MFFD) network that employs a focus-diffuse transmission pathway to preserve fine-grained spatial details from high-resolution layers and propagate them to semantic features; an efficient CS-Head detection architecture that utilizes parameter-sharing convolution to enable efficient processing on embedded platforms; and an optimized loss function combining Normalized Wasserstein Distance (NWD) with InnerCIoU to improve localization accuracy for small targets. Experimental validation on the VisDrone2019 dataset demonstrates that RMH-YOLO achieves a precision and recall of 53.0% and 40.4%, representing improvements of 8.8% and 7.4% over the YOLOv8n baseline. The proposed method attains mAP50 and mAP50:95 of 42.4% and 25.7%, corresponding to enhancements of 9.2% and 6.4%, respectively, while maintaining computational efficiency with only 1.3 M parameters and 16.7 G FLOPs. Experimental results confirm that RMH-YOLO effectively improves small-target detection accuracy while maintaining computational efficiency, demonstrating its broad application potential in diverse UAV aerial monitoring scenarios.

## 1. Introduction

Unmanned Aerial Vehicle (UAV) vision systems have emerged as critical platforms for aerial monitoring and remote sensing applications across diverse domains. The integration of high-resolution imaging capabilities with UAV platforms enables comprehensive spatial data acquisition for various applications, including urban monitoring, environmental surveillance, infrastructure inspection, and emergency response [1,2,3,4,5,6,7,8]. These applications leverage the unique capabilities of airborne platforms—including wide-area coverage, flexible deployment, and high-resolution imaging—to address monitoring challenges that are difficult or impractical for ground-based systems.

However, small-target detection in UAV aerial imagery faces critical challenges arising from the inherent characteristics of high-altitude imaging and complex environmental conditions. First, the limited spatial resolution relative to flight altitude results in small targets occupying minimal pixel representation (often fewer than 32 × 32 pixels), making it difficult for detection models to extract discriminative features from low-resolution imagery [9]. Second, the dynamic nature of UAV flight—with frequent altitude and viewing angle changes—introduces substantial scale variations in captured imagery, requiring models to maintain robust feature representation across diverse perspectives [10]. Third, the complex backgrounds encountered in aerial scenarios, containing diverse interfering elements such as vegetation, urban structures, and terrain variations, readily cause confusion between targets and background [11]. Fourth, varying illumination conditions—including intense sunlight, shadows, and low-light environments—significantly affect imaging quality and degrade the discriminability of target features [12]. These challenges collectively limit the performance of detection algorithms when processing UAV aerial imagery.

To address these challenges specific to small-target detection in UAV aerial imagery, this study proposes RMH-YOLO, a refined multi-scale architecture with targeted improvements optimized for processing small objects in UAV-captured scenes. The model integrates four key innovations:

- First, a Refined Feature Module (RFM) is integrated into the backbone network to enhance feature extraction from low-resolution imagery. By fusing channel and spatial attention mechanisms within residual connections, the RFM dynamically recalibrates multi-scale feature representations, improving the discriminative capability for small targets while maintaining contextual integrity for larger instances.
- Second, a Multi-scale Focus-and-Diffuse (MFFD) network is designed to optimize feature propagation and fusion for aerial imagery. The MFFD employs a dual-phase strategy: a focus stage that aggregates high-resolution spatial cues from shallow feature layers with semantic context from deeper features, followed by a diffuse stage where enriched features are adaptively distributed across hierarchical levels to enhance representational capacity for small objects.
- Third, an efficient detection head (CS-Head) utilizing parameter-sharing convolution is proposed to enable efficient processing on embedded UAV platforms. By sharing convolution kernels across detection tasks while maintaining discriminative capability through scale-specific adjustments, the CS-Head reduces computational requirements suitable for resource-constrained deployment.
- Fourth, an optimized loss function combining Normalized Wasserstein Distance (NWD) with InnerCIoU is employed to improve localization accuracy for small targets in aerial imagery, addressing the challenge of precise bounding box regression for objects with minimal pixel representation.

## 2. Related Work

### 2.1. Generic Target Detection Methods

Object detection, a central problem in computer vision, has advanced significantly in methodology over recent years. Modern detectors are broadly classified into two types: two-stage and single-stage architectures, each with distinct representational characteristics and computational trade-offs [13,14,15].

Two-stage detectors, such as the R-CNN series [13,14], adopt a sequential refinement approach. These methods first generate class-agnostic region proposals, which are subsequently classified and regressed via region-wise feature extraction. This design enhances recognition accuracy by filtering out much of the background and concentrating computation on promising regions. However, the multi-stage process inherently increases computational cost and constrains inference speed.

In comparison, single-stage detectors perform classification and bounding-box regression directly from dense feature maps in a single feed-forward pass. Representative models include SSD [15], RetinaNet [16], and the YOLO family [17]. Among these, YOLO-based detectors have gained broad adoption in practical applications due to their favorable accuracy–efficiency balance. Unlike the anchor-based mechanism used in SSD, YOLO employs a grid-based strategy: the image is divided into a fixed set of non-overlapping cells, each responsible for predicting objects whose centers fall within it. This unified and center-aware formulation facilitates end-to-end training and improves inference efficiency by eliminating explicit region proposal generation [18].

### 2.2. YOLO-Based Algorithm Improvements for UAV Aerial Small-Target Detection

UAV aerial small-target detection confronts unique challenges: objects with extremely small scales (typically less than 5% of the image), low pixel resolution of targets, and cluttered backgrounds (such as overlapping vegetation and undulating terrain). Due to its favorable balance between accuracy and real-time performance, the YOLO framework has become a primary choice for adaptation, with research focusing on two core directions: strengthening the representation of small-target features and optimizing model efficiency for UAV platforms.

A key emphasis in feature enhancement is modifying YOLO’s Neck network, which is crucial for cross-scale feature fusion. Zhao et al. [19] enhanced the feature fusion mechanism of the neck network by introducing a BiFPN. Luo et al. [20] proposed a UAV-tailored multi-scale FPN with optimized lateral connections, integrating low-level high-resolution features (for small-target localization) and high-level semantic features (for category discrimination) to alleviate the dilution of small-object features.

Similarly, Hui et al. [21] incorporated an attention mechanism into the NECK, adaptively weighting multi-scale feature channels to prioritize small-target information and suppress background noise. Wang et al. [22] further embedded a one-to-many feature fusion mechanism into the NECK, enriching the propagation of small-target features.

Beyond optimizations of the Neck Network, improvements to the Backbone network targeting fine-grained small-target features have also been explored. For the core C2f module of YOLOv8, Bi et al. [23] replaced standard convolutions with depthwise separable convolutions and added local attention. This dual optimization enables the Backbone to retain more spatial details of small targets while reducing redundant computations, balancing feature preservation and efficiency for UAV deployment. Liao et al. [24] utilized multi-scale heterogeneous convolutional kernels to enhance channel and spatial attention within C2f, boosting spatial perception capabilities. Wei. [25] introduce the receptive field network block leverages the receptive field convolutional block attention to capture fine-grained local details.

## 3. Method

### 3.1. Overview of RMH-YOLO

**Figure 1:**
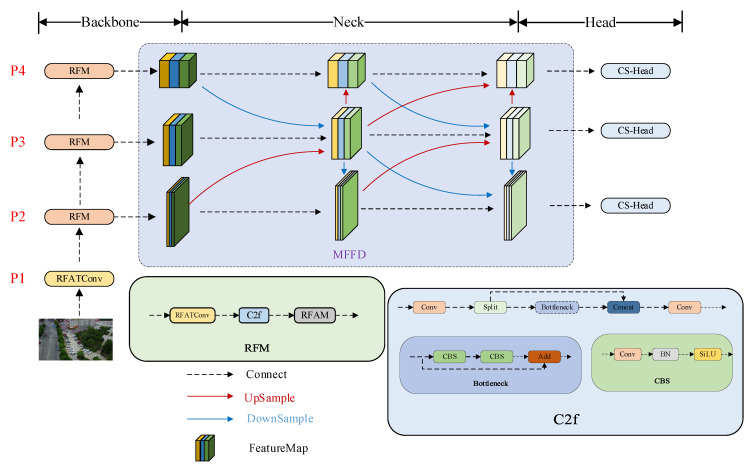
Structure of RMH-YOLO model.

Detecting small objects in unmanned aerial vehicle (UAV) imagery remains particularly challenging owing to the minimal spatial extent of such targets—typically spanning only 5–20 pixels—coupled with their low semantic salience and high susceptibility to occlusion within complex environments, including dense vegetation, shadowed regions, and structured urban settings. Conventional convolutional architectures tend to suppress subtle yet discriminative features during background suppression, thereby diminishing the representational fidelity of small objects. To address this shortcoming, we propose the RMH-YOLO model. The structure of the RMH-YOLO model is depicted in Figure 1.

To tackle the inherent limitation of insufficient small-target feature extraction in prior backbone networks—particularly critical for UAV aerial scenarios where small targets suffer from low pixel occupancy and weak feature expression—we innovatively introduce the Refined Feature Module (RFM), which embeds both spatial and channel attention mechanisms to adaptively emphasize task-relevant spatial regions (e.g., local details of small targets) and feature channels (e.g., discriminative cues for tiny objects), thereby enhancing the model’s ability to capture overlooked small-target features; we further present the Multi-scale Focus-and-Diffuse (MFFD) network to address shallow information loss in traditional neck structures (shallow features being essential for small-target localization yet often diluted during multi-scale fusion), leveraging its unique “focus-diffuse” path to first preserve fine-grained shallow details and then propagate them to higher-scale maps for effective information compensation; for the detection head, to resolve parameter redundancy in YOLOv8’s original design (a constraint for UAV on-edge deployment due to increased computational cost without proportional gains), we design the lightweight Convolution-Shared Head (CS-Head), which uses shared parameter convolution across tasks and scales to eliminate redundancy while maintaining detection capability, ultimately achieving model lightweighting.

### 3.2. Refined Feature Module

**Figure 2:**
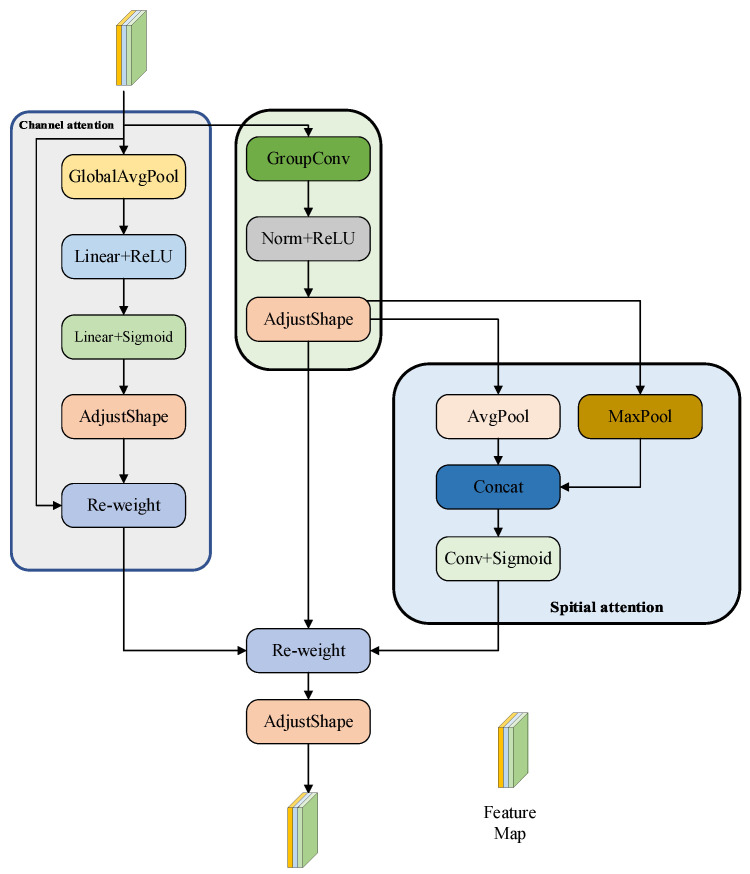
Structure of RFATConv.

Small object detection in images captured by unmanned aerial vehicles (UAVs) remains a challenging task, primarily due to the extremely limited pixel footprint of targets (often between 5 to 20 pixels), their low semantic saliency, and frequent occlusion amidst cluttered backgrounds—such as vegetation, shadows, and urban structures. Conventional convolutional neural networks often fail to retain critical fine-grained details while filtering out complex background noise, resulting in degraded feature quality for such small objects. To mitigate this issue, we propose a novel convolutional module termed RFATConv (see Figure 2), which employs grouped convolutions combined with a dual-attention mechanism to enhance feature selectivity and discrimination capability for small targets.

RFATConv’s efficacy stems from three synergistic components:

First, grouped convolution with $groups=C_{in}$ processes each channel independently via a k×k kernel, mapping input $X\in R^{B\times C_{in\;}\times H\times W}$ to an expanded feature map $F_{exp}\;\in R^{B\times C_{in\;}\times k^{2}\times H\times W}$. This preserves k×k local receptive field details (e.g., small-target edges) that standard convolution dilutes, with padding ensuring spatial resolution remains H×W.

Second, spatial attention refines $F_{exp}$ by generating weights:

(1) $$w_{rf}\;=Sigmoid(Conv(Concat(\max(F_{\exp}\;),mean(F_{\exp\;}))))$$

emphasizing target-related local regions.

Third, channel attention computes weight:

(2) wch=Sigmoid(FC2(ReLU(FC1(GAP(X)))))

to amplify discriminative channels (e.g., edge-carrying ones) while suppressing background-dominated ones. These components collaborate to tackle UAV small-target challenges: grouped convolution retains critical local details, channel attention filters noisy channels, and spatial attention focuses on target-relevant regions. Their integration ensures $F_{rf-att}=(F_{\exp}⊙w_{ch})⊙w_{rf}$ enhances small-target features amid clutter, making RFATConv robust to low resolution and complex backgrounds in UAV scenarios.

**Figure 3:**
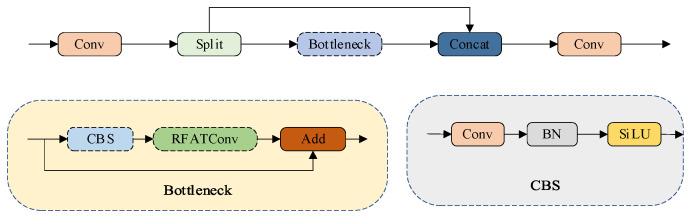
Structure of RFAM Module.

To enhance the representational capacity of small-target features within the backbone architecture while maintaining computational efficiency, we propose a Refined Feature Module (RFM). Illustrated schematically in Figure 1, the RFM adopts an integrated design consisting of:

(3) RFM=RFATConv+C2f+RFAM

where RFAM denotes the modified bottleneck within C2f. RFAM is depicted in Figure 3. This design inherits the efficient multi-scale feature transmission of YOLOv8’s backbone while strengthening small-target feature preservation and refinement.

In the downsampling stage of the backbone, we replace the conventional convolution in YOLOv8’s Conv with RFATConv. Traditional convolution-based downsampling often causes loss of shallow small-target features due to aggressive spatial compression, which is critical for UAV aerial scenarios where small targets already have limited pixel representation. By contrast, RFATConv’s grouped convolution preserves k×k local receptive field details of small targets, while its dual attention mechanisms enhance discriminative features before downsampling.

To further strengthen small-target features in high-level feature maps, we introduce RFAM by embedding RFATConv into the second convolution of C2f’s bottleneck. In YOLOv8’s original C2f, bottlenecks use two standard convolutions for feature transformation, which lack adaptive refinement for small targets. RFAM replaces the second convolution with RFATConv, enabling the module to perform fine-grained optimization of features after initial transformation. Thus, RFAM strengthens critical features without increasing computational burden, making it suitable for resource-constrained UAV platforms.

### 3.3. Multi-Scale Feature Fusion Network

#### 3.3.1. ADSF Method

The backbone network produces features at multiple scales—designated as P2, P3, and P4—each conveying unique information. Among these outputs, P2 corresponds to a shallow layer with high spatial resolution, preserving fine details such as edges and textures of small objects, though it contains relatively limited semantic content. On the other hand, P4, derived from a deeper stage, possesses a larger receptive field and offers richer semantic representations that are advantageous for object recognition, despite its lower spatial accuracy. *P*3 serves as an intermediate layer that balances detail and semantics, yet still falls short of fully leveraging the complementary strengths of both finer and coarser features. Relying solely on a single scale—such as only *P*4 or *P*2—can lead to substantial information loss. For example, the coarse resolution of *P*4 may result in undetected small objects, while the weak semantics of *P*2 could increase the risk of misclassification. Hence, it is crucial to effectively integrate information from all three hierarchical levels to synergize their spatial and semantic advantages for improved detection performance.

**Figure 4:**
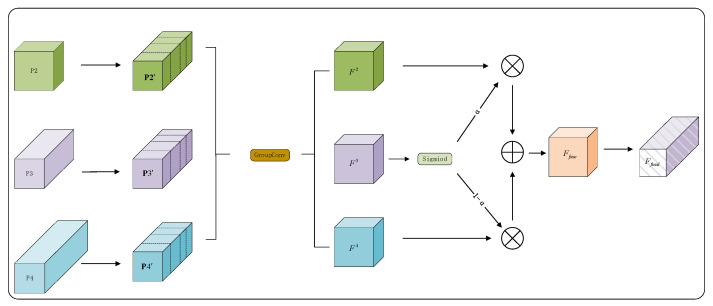
Structure of ADSF Method.

To address these limitations, we propose the Adaptive Dynamic Scale-Focused Fusion (ADSF) module, illustrated in Figure 4. This mechanism adaptively recalibrates and combines multi-level features via dynamic weighting, enabling a more efficient fusion of information across different scales.

Its core workflow are as follows:

To align multi-scale features, P2 (high resolution) is downsampled to the resolution of P3 using dilated convolution (stride = 2, dilation = 2), which preserves receptive field coverage. P4 (low resolution) is upsampled via bilinear interpolation after its channel dimension is adjusted with a 1 × 1 convolution. Meanwhile, P3 (reference scale) only undergoes channel projection via the same 1 × 1 convolution. Let $C_{out}$ represent the target number of channels; the aligned feature maps P2,P3,P4 all share the shape $R^{B\times C_{out\;}\times H_{3\;}\times W_{3}}$, where $H_{3}$ and $W_{3}$ denote the spatial dimensions of P3.

These aligned features are subsequently processed through a grouped convolution (GroupConv) module. This design reduces both parameter count and computational cost while enabling cross-scale feature interaction at the channel level. The outputs of this operation are denoted as $F^{2},F^{3},\;F^{4}$. A dynamic attention coefficient αis then derived from $F^{3}$ A dynamic attention weight α is calculated using $F^{3}$ and a sigmoid activation:

(4) $$\alpha =\sigma (F^{3})=\frac{1}{1+e^{-F^{3}}},$$

where α∈[0,1] serves to adaptively modulate the contributions between the semantically rich $F^{4}$ and the spatially detailed $F^{2}$. The final fused representation is computed via element-wise multiplication and summation:

(5) $$F_{fus}=\alpha ⊙F^{4}+(1-\alpha )⊙F^{2}$$

where ⊙ indicates the Hadamard product. The fused feature $F_{fuse}$ effectively integrates complementary information from P2, P3 and P4, and is further utilized for downstream processing.

In brief, the ADSF method fully leveragea the complementary information of multi-scale feature maps (P2, P3, P4) extracted by the backbone and addresses the limitations of single-scale feature representation. ADSF integrates group convolution for efficient channel interaction and adaptive weighting for dynamic feature fusion, aiming to achieve effective and lightweight multi-scale feature integration.

#### 3.3.2. MFFD Network

The Path Aggregation Feature Pyramid Network (PAFPN) in YOLOv8 adopts a bidirectional feature propagation mechanism across hierarchical layers. While this design enhances the integration of multi-scale context to a certain degree, it exhibits a notable structural deficiency: during both the top-down and bottom-up information flows, high-resolution spatial details—particularly from early-stage feature maps such as P2, which are critical for accurately locating small objects—are gradually diluted. Such attenuation of fine-grained cues significantly compromises the detection performance for small targets in environments with wide-ranging scale distributions.

To overcome this limitation, we introduce a Multi-Scale Focus Fusion-Diffusion (MFFD) module, the overall structure of which is presented in Figure 1.

The MFFD module begins by integrating multi-level inputs (P2, P3, P4) using an Adaptive Dynamic Scale-Focused Fusion (ADSF) strategy. ADSF learns weighting coefficients across scales in a data-dependent manner, thereby preserving both high-frequency spatial cues from early layers and semantically rich representations from deeper layers during the fusion process.

To propagate shallow-level details downward, a 3 × 3 convolutional layer is applied, while deep semantic information is transmitted upward via an upsampling operator. The resulting feature streams are then concatenated with the original P2 and P4 layers, respectively. Throughout this process, the ADSF module is employed to perform the initial phase of feature diffusion. By dynamically modulating fusion weights across scales in real time, ADSF ensures that both high-resolution details and high-level semantics are effectively combined during concatenation, thereby minimizing interference and preserving informational integrity.

After completing the first feature diffusion, the MFFD network repeats the aforementioned operations. Specifically, the 3 × 3 convolution and upsampling modules that participated in the first stage of feature diffusion are concatenated with the subsequent corresponding outputs. This repetition realizes the second stage of feature diffusion. Throughout this repeated process, ADSF continuously exerts its function of dynamically balancing and fusing multi-scale information. As a result, each detection scale of the model is endowed with detailed contextual information, and the mutual complementation of shallow details and deep semantics is achieved. This not only strengthens the model’s representation of multi-scale features but also significantly improves its detection effect on targets of different scales, especially for small targets that were previously difficult to detect accurately due to shallow feature attenuation.

### 3.4. CSLD Detection Head

**Figure 5:**
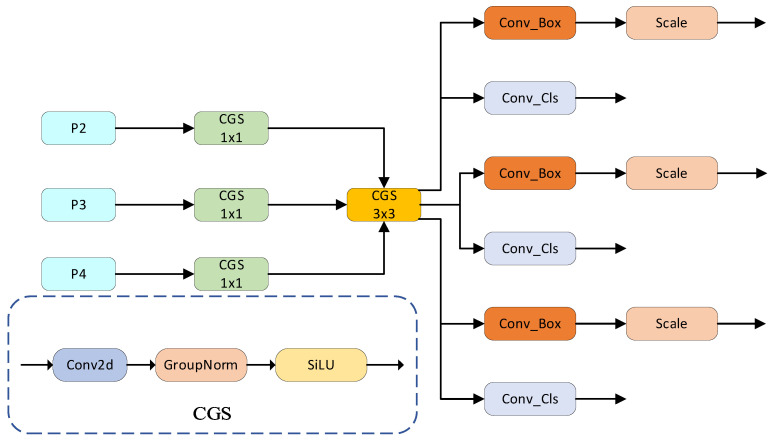
CS-head Detection Head structure diagram.

The original YOLOv8n detection head suffers from two primary limitations: high computational complexity and inadequate exploitation of multi-scale feature representations. To mitigate these issues, we introduce a lightweight alternative termed the CS-Head, the structure of which is illustrated schematically in Figure 5.

CS-head integrates three key design strategies: First, it employs Group Normalization (GN) for feature normalization, which, unlike Batch Normalization (BN), operates independently of batch size. This ensures stable feature distribution regularization across varying batch scales, thereby enhancing localization and classification performance. Second, a shared convolution module is introduced to eliminate redundant parameters caused by independent convolution operations in multiple detection branches, significantly reducing model size and improving efficiency for resource-constrained devices. Third, a Scale layer is incorporated to adjust shared convolution outputs, addressing scale mismatches between shared features and diverse target sizes detected by different heads. These synergistic designs enable CS-head to reduce parameters and computational load while minimizing accuracy degradation, achieving an optimal balance between lightweight architecture and detection performance for efficient deployment in practical scenarios

### 3.5. Loss Function Design

In object detection, the loss function significantly influences the precision of bounding box regression. The Complete Intersection over Union (CIoU) loss enhances the conventional IoU-based loss by incorporating geometric constraints. Its formulation is given as:

(6) $$Loss_{CIoU}=1-IoU+\frac{d^{2}}{c^{2}}+\alpha \nu$$

In this context, IoU measures the extent of overlap between predicted and actual bounding boxes. The distance, denoted as d, corresponds to the Euclidean separation of the center coordinates of both boxes, whereas c indicates the diagonal measurement of the minimal enclosing rectangle that encompasses the two regions—serving to penalize deviations in center alignment; the weight coefficients α and ν, which measure the similarity in aspect ratios between the two boxes, further regulate the regression process. The calculation formulas for α and ν are, respectively:

(7) $$\alpha =\frac{\nu }{\left(1-IoU\right)+\nu }$$

(8) $$\nu =\frac{4}{\pi^{2}}{\left(\arctan\frac{\omega^{gt}}{h^{gt}}-\arctan\frac{\omega }{h}\right)}^{2}$$

Here, $\omega^{gt}$ and $h^{gt}$ denote the width and height of the ground truth bounding box, while ω and h correspond to the width and height of the predicted box.

Although CIoU loss improves bounding box regression and convergence by incorporating center-point distance and aspect ratio constraints, it exhibits limited generalization across diverse detection tasks. In particular, for small-target detection in UAV imagery—where objects often occupy very few pixels and possess weak features—CIoU demonstrates notable limitations in both accuracy and efficiency. It struggles to deliver the high-precision localization performance required for robust small-target detection in complex aerial environments.

The InnerCIoU loss function enhances bounding box regression for small targets in UAV imagery by adaptively rescaling auxiliary detection boxes through a scale factor ratio. This method reconstructs and refines the geometric relationships between predicted and ground truth boxes. The detailed computational procedure is outlined below:

First, the parameters of the auxiliary box are defined based on the predicted box and the ground truth box. The predicted box is represented by its center coordinates (x,y), the width is ω, and the height is h; the center point coordinates of the ground truth box are $\left(x^{gt},y^{gt}\right)$, the width is $\omega^{gt}$, and the height is $h^{gt}$. Using the scale factor ratio, the dimensions of the ground truth box are adaptively adjusted to derive the auxiliary box parameters used in the computation:

(9) $$b_{l}^{gt}=x_{c}^{gt}-\frac{w^{gt}\times ratio}{2},b_{r}^{gt}=x_{c}^{gt}+\frac{w^{gt}\times ratio}{2}$$

(10) $$b_{t}^{gt}=y_{c}^{gt}-\frac{h^{gt}\times ratio}{2},b_{b}^{gt}=y_{c}^{gt}+\frac{h^{gt}\times ratio}{2}$$

(11) $$b_{l}=x_{c}-\frac{w\times ratio}{2},b_{r}=x_{c}+\frac{w\times ratio}{2}$$

(12) $$b_{t}=y_{c}-\frac{h\times ratio}{2},b_{b}=y_{c}+\frac{h\times ratio}{2}$$

Next, calculate the intersection (inter) and union (union) of the auxiliary box and the predicted box:

(13) $$\begin{array}{c} inter=(min(b_{r}^{gt},b_{r})-max(b_{l}^{gt},b_{l}))\times (min(b_{b}^{gt},b_{b})-max(b_{t}^{gt},b_{t})) \end{array}$$

(14) $$union=(w^{gt}\times h^{gt})\times {\left(ratio\right)}^{2}+(w\times h)\times {\left(ratio\right)}^{2}-inter$$

On this basis, calculate the improved IoU ($IoU^{inner}$):

(15) $$IoU^{inner}=\frac{inter}{union}$$

(16) $$\begin{array}{c} L_{InnerCIoU}=L_{CIoU}+IoU-IoU^{inner} \end{array}$$

**Figure 6:**
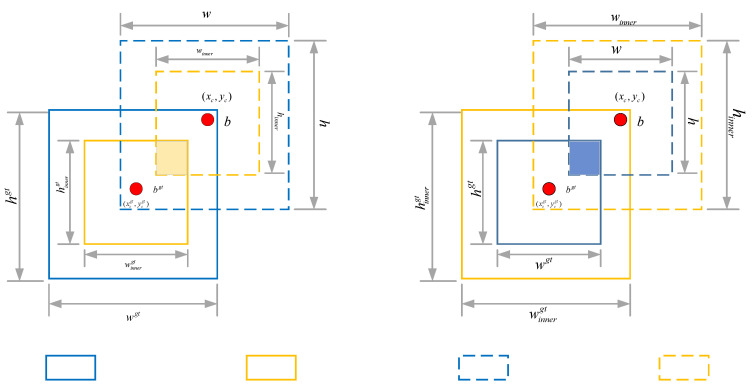
InnerIoU loss function schematic diagram.

As illustrated in Figure 6, the schematic of the InnerIoU loss function demonstrates that when the ratio value falls below 1, the auxiliary box becomes smaller than the ground truth box. This reduction in size diminishes the overlap between the two boxes, thereby lowering the Intersection over Union (IoU) value during regression. Under this condition, the absolute gradient magnitude increases relative to the baseline, accelerating convergence of the regression process. Conversely, when the ratio exceeds 1, a larger auxiliary bounding box expands the regression scope, thereby improving localization performance for targets with relatively ambiguous or soft CIoU characteristics. Hence, in the context of small-target detection in UAV aerial imagery, employing the InnerCIoU loss with a ratio below 1 not only expedites bounding box regression but also enhances positioning accuracy in complex environments.

As discussed, InnerCIoU improves detection performance for UAV small targets by adaptively scaling auxiliary bounding boxes. However, since InnerCIoU builds upon CIoU, it inherits a key limitation: CIoU is highly sensitive to minor positional deviations. Even slight misalignments between predicted and ground truth boxes can trigger sharp decreases in IoU and induce instability in the loss function. This problem becomes particularly severe in UAV-based small-object detection, where targets are tiny and localization noise is common, often leading to training divergence and ultimately impairing detection accuracy.

To overcome these challenges, we incorporate Normalized Wasserstein Distance (NWD) into the regression objective. NWD measures similarity between bounding boxes using the normalized Wasserstein distance, which offers greater robustness to small coordinate deviations. The NWD loss is defined as follows:

(17) $$NWD\left(N_{a},N_{b}\right)=\exp\left(-\frac{\sqrt{W_{2}^{2}\left(N_{a},N_{b}\right)}}{C}\right)$$

where $W_{2}^{2}(N_{a},N_{b})$ is the second-order Wasserstein distance and C is the number of categories. The second-order Wasserstein distance $W_{2}^{2}(N_{a},N_{b})$ is calculated as:

(18) $$W_{2}^{2}(N_{a},N_{b})={\left\|\left({\left[cx_{a},cy_{a},\frac{w_{a}}{2},\frac{h_{a}}{2}\right]}^{T},{\left[cx_{b},cy_{b},\frac{w_{b}}{2},\frac{h_{b}}{2}\right]}^{T}\right)\right\|}_{2}^{2}$$

NWD reformulates the deviation between predicted and ground-truth bounding boxes as a continuous probability-based distance measure. This approach proves especially advantageous in aerial image analysis, where subtle localization errors are common—particularly in UAV-based small object detection scenarios. By offering a smoothly differentiable loss function, NWD promotes stable gradient propagation and enhances convergence behavior during model optimization.

We integrate InnerCIoU with Normalized Wasserstein Distance (NWD), leveraging the complementary advantages of both methods. InnerCIoU complements this process by adaptively adjusting the scale of auxiliary bounding boxes specifically for small targets. While InnerCIoU enhances regression precision in UAV aerial images, NWD ensures training stability by mitigating loss volatility caused by small coordinate deviations. Together, they facilitate progressive optimization of detection accuracy: NWD maintains stable gradient flow, while InnerCIoU refines bounding box localization. The synergy of these methods results in improved robustness and detection performance for small targets in complex aerial scenarios.

## 4. Experiment

### 4.1. Data Set and Experimental Setup

**Figure 7:**
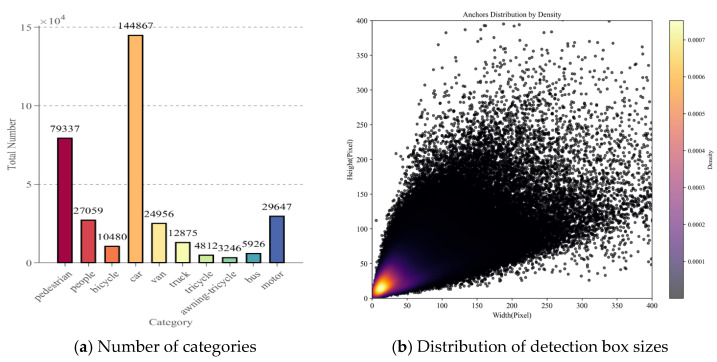
Statistical results of the VisDrone2019 dataset.

In this study, we employ the widely used VisDrone2019 dataset—a publicly available benchmark for drone-based visual object detection, originally curated by the AISKYEYE group at Tianjin University. The dataset comprises 8629 fully annotated images capturing a range of urban and rural traffic environments, with ground sample distances (GSDs) varying between 0.02 and 0.1 m per pixel. It includes ten object categories, including “pedestrian” and “people,” among others. The images are partitioned in a 7:2:1 ratio into training, validation, and test subsets, containing 6471, 548, and 1610 images, respectively. The class distribution across the training subset is depicted in Figure 7.

**Figure 8:**
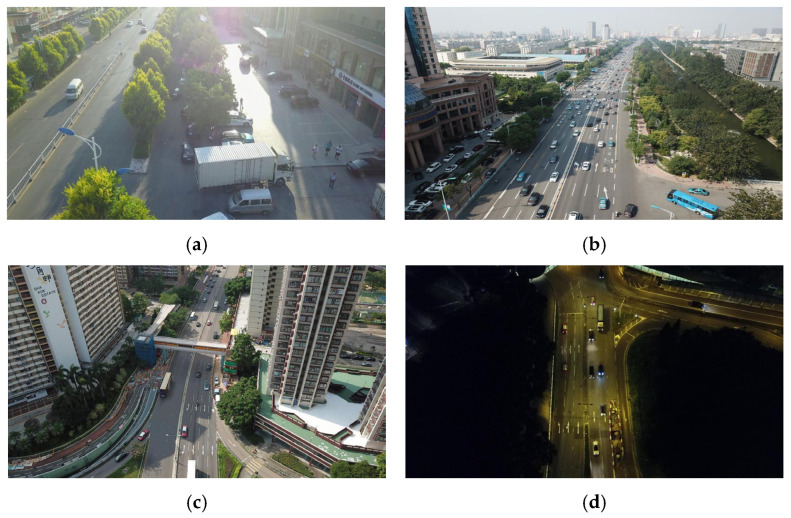
Images of typical scenarios in the VisDrone-2019 dataset. (**a**) Complex background. (**b**) Multi-scale targets. (**c**) Small target. (**d**) Night scene.

Figure 7 presents the statistics of the VisDrone2019 dataset. Figure 7a displays the quantity distribution of various targets in the training set. There are significant differences: car has the largest number (144,867), pedestrian ranks second (79,337), and awning-tricycle is relatively few (3246). This imbalance reflects real-world target occurrence frequency differences and challenges model performance balance across categories, so subsequent training and optimization must consider this. Figure 7b shows the pixel distribution of detection box height and width, with color brightness representing instance density (brighter means denser). Detection boxes are concentrated in low-width (0–100 pixels) and low-height (0–200 pixels) ranges, and brighter colors here indicate dense small-size instances. This reveals that small-scale targets occupy a large proportion, requiring models to have strong small-target detection capabilities. Figure 8 displays aerial images in typical scenarios:

Figure 8a is a complex street scene with urban roads, buildings, and vegetation. Targets range from large trucks to small pedestrians; the background has intertwined roads, trees, and buildings, interfering with detection; illumination has light-shadow contrasts and uneven brightness, increasing target-background distinction difficulty. Figure 8b is an urban arterial road scene with multi-scale targets (large buses to small motorcycles) and a complex background (road facilities, buildings, green belts); daytime illumination has unevenness and road reflections, making conditions non-ideal. Figure 8c shows a road between high-rises with small targets (vehicles with insufficient pixel features) and a complex background (buildings, bridges, vegetation); daytime illumination has building shadows, affecting small-target detection. Figure 8d is a night-time road scene: vehicle targets have low contrast and small effective features due to low brightness and light highlights; the dark background compresses visual info, and complex local illumination (vehicle lights, road reflections) increases feature extraction difficulty.

### 4.2. Experimental Environment and Evaluation Metrics

All experiments were conducted in a PyTorch 2.1.0 environment with Python 3.10 and CUDA 12.1. The hardware platform consisted of a 12th Gen Intel Core i5-12400F CPU and an NVIDIA GeForce RTX 3090 GPU with 24 GB of memory. To ensure experimental reproducibility and eliminate the influence of pre-trained parameters, all models were trained from scratch without using any pre-trained weights. Key hyperparameters included the use of a Stochastic Gradient Descent (SGD) optimizer with an initial learning rate of 0.01 and a momentum coefficient of 0.937. Input images were resized to 640 × 640 pixels, and the model was trained for 200 epochs with a batch size of 8.

Model performance was evaluated using standard object detection metrics, including Precision (P), Recall (R), and mean Average Precision (mAP). mAP50 refers to the mean average precision calculated at an intersection-over-union (IoU) threshold of 0.5, reflecting detection accuracy under a moderate localization criterion. mAP50:95 represents the average precision computed over multiple IoU thresholds ranging from 0.5 to 0.95 (in increments of 0.05), providing a comprehensive performance measure across varying localization strictness:

(19) $$P=\frac{TP}{TP+FP}$$

(20) $$R=\frac{TP}{TP+FN}$$

(21) $$AP=\int_{0}^{1}P(r)dr$$

(22) $$mAP=\frac{1}{K}\sum_{j=1}^{K}AP_{j}$$

True positives (TP) refer to positive instances that are both correctly localized and classified. False positives (FP) denote negative samples erroneously identified as positive, while false negatives (FN) correspond to undetected or misclassified positive instances.

Precision measures the proportion of correctly predicted positives among all detections classified as positive, reflecting the reliability of positive predictions. Recall evaluates the fraction of true positives successfully detected among all actual positive samples, indicating the model’s ability to identify targets exhaustively.

Average Precision (AP) integrates the precision-recall curve to evaluate detection performance for a single category. The mean Average Precision (mAP) averages AP values across all classes, offering a holistic assessment of the model’s accuracy over the entire dataset.

To quantify computational efficiency, three metrics are employed: parameter count (Params), floating-point operations (FLOPs), and frames per second (FPS). These reflect model size, computational complexity, and real-time inference capability, respectively. Specifically, Params indicate the number of trainable parameters; FLOPs measure arithmetic operations per forward pass; and FPS quantifies processing speed in images per second.

### 4.3. Ablation Experiments

#### 4.3.1. RMH-YOLO Ablation Experiments

To evaluate the detection capability of the proposed model and examine the individual contribution of each enhanced component along with its synergistic impact on overall performance, a series of ablation studies was performed using YOLOv8n as the baseline. Groups B–D were designed to independently assess the efficacy of specific modifications: RFAT for refining the backbone network, MFHD as a replacement in the neck network, and CSLD substituted into the detection head. Subsequently, Groups E–H involved progressively stacking these modules to analyze collaborative performance gains between components. The experimental configurations are documented in Table 1.

The ablation experimental results are shown in Table 2

From the experimental results of Groups B, C, and D with individual module improvements:

Incorporating the RFAT backbone in Group B yields consistent improvements across detection metrics relative to the baseline (Group A), with gains of 2.5% in precision, 2.1% in recall, 2.5% in mAP50, and 1.8% in mAP50:95. These results suggest that RFAT enhances discriminative feature extraction and provides more informative representations for subsequent detection stages.

Replacing the original neck with the proposed MFHD module in Group C leads to more substantial gains: precision, recall, mAP50, and mAP50:95 increase by 5.2%, 6.0%, 6.8%, and 4.6%, respectively. The MFHD module demonstrates a pronounced ability to integrate multi-scale contextual features and improve semantic consistency across scales.

In Group D, substitution with the CSLD detection head maintained nearly equivalent precision, recall, mAP50, and mAP50:95 relative to the baseline. Notably, computational load decreased by 1.6 GFLOPs and frame rate improved by 17.0 FPS. This demonstrates that CSLD effectively streamlines computation and accelerates inference while preserving model accuracy.

From the experiments of Groups E, F, G, and H with module stacking:

Relative to the baseline (Group A), the model in Group E demonstrates an 8.8% improvement in mAP50 and a 5.9% gain in mAP50:95. These enhancements exceed those achieved by models utilizing either RFAT or MFHD individually (Groups B and C), indicating a synergistic interaction between the two modules that amplifies both feature representation and cross-scale integration. The refined backbone features are thus more comprehensively exploited by the neck structure, substantially boosting detection performance. Notably, these accuracy improvements entail elevated computational costs: FLOPs increase by 13.5 G (from 8.1 G to 21.6 G). These results underscore a critical trade-off between detection accuracy and operational efficiency in the design of deep learning-based detectors.

Compared with Group E, in Group F, mAP50 slightly decreases from 42.0% to 41.6%, a slight decrease of 0.4%; mAP50:95 slightly decreases from 25.2% to 25.1%, a slight decrease of 0.1%; precision (P) and recall rate (R) also slightly decrease. FLOPs decrease from 21.6 G to 16.7 G, a decrease of 4.9 G; FPS increases from 91.5 to 76.6, an increase of 14.9; the number of parameters decreases from 1.8 M to 1.3 M. This shows that the introduction of CSLD can reduce the computational cost while keeping the model performance stable.

In Group G, on the basis of Group F, InnerCIoU is stacked, and mAP50 increases by 0.3% compared with Group F, and mAP50:95 increases by 0.4%. It shows that InnerCIoU, as a loss function, can optimize the regression performance of detection boxes and improve the false detection and missed detection phenomena of the model.

Compared with Group G, in Group H, mAP50 is further increased by 0.5% from 41.9% to 42.4%; mAP50:95 is increased by 0.2% from 25.5% to 25.7%; precision (P) and recall rate (R) are also slightly increased. FLOPs, the number of parameters basically the same as those of Group G, indicating that NWD can work synergistically with InnerCIoU to further optimize detection performance, especially in complex scenarios. The improvement of detection precision for small targets or difficult-to-distinguish targets is more obvious.

Compared with Group A, in Group H, mAP50 is increased from 33.2% to 42.4%, an increase of 9.2%; mAP50:95 is increased from 19.3% to 25.7%, an increase of 6.4%; precision is increased from 44.2% to 53.0%, an increase of 8.8%; the recall rate is increased from 33.0% to 40.4%, an increase of 7.4%, fully reflecting the significant promotion effect of stacking each module on the overall detection performance of the model.

#### 4.3.2. Neck Network Ablation Experiments

From the analysis of the ablation tests on all modules above, it is known that the replacement method using the neck network brings the greatest performance improvement to the overall model. To verify the effectiveness of each method in the multi-scale feature fusion network of the neck structure proposed in this paper, the PAFPN used in YOLOv8 is taken as the benchmark method for the neck network, and the following improvements are made in sequence:

Neck_1: The PAFPN neck network in YOLOv8n.

Neck_2: Add a *P*2 detection head.

Neck_3: Remove the *P*5 down-sampling module in the backbone network, add a *P*2 detection head, and remove the *P*5 detection head.

Neck_4: Replace the neck structure of Neck_3 with the MFHD network structure.

**Figure 9:**
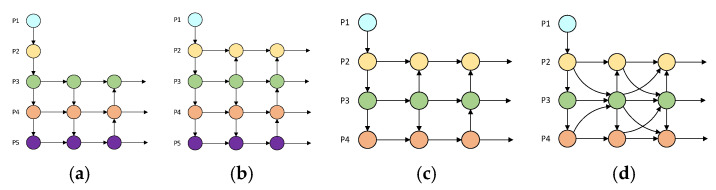
Different neck network structure diagrams. (**a**) Neck_1; (**b**) Neck_2; (**c**) Neck_3; (**d**) Neck_4.

The structural diagrams of these different neck networks are illustrated in Figure 9.

The results of the neck network ablation tests are shown in Table 3.

Conclusions drawn from the ablation experiment results are as follows:

Neck_2: Based on Neck_1 (the PAFPN neck network in YOLOv8n), a *P*2 detection head is added. The precision (47.7%), recall rate (35.5%), mAP50 (36.7%), and mAP50:95 (21.8%) are all higher than those of Neck_1 (44.2%, 33.0%, 33.2%, 19.3%). However, the computational load increases from 8.1 to 12.2, and the number of parameters slightly decreases from 3.0 M to 2.9 M. This reflects that adding a *P*2 detection head brings benefits to small-target detection, and the model complexity does not increase significantly.

Neck_3: The *P*5 down-sampling module and detection head in the backbone network are removed, and a *P*2 detection head is added. The precision (44.7%) is slightly higher than that of Neck_1, and the recall rate (35.9%), mAP50 (35.8%), and mAP50:95 (21.2%) are all improved. The computational load (10.4) is higher than that of Neck_1 but lower than that of Neck_2, and the number of parameters (1.0 M) is greatly reduced, achieving a certain balance between performance and lightweight.

Comparison between Neck_3 and Neck_2: The precision and mAP-related indicators of Neck_3 are slightly lower than those of Neck_2, but the recall rate is better. Moreover, the computational load and number of parameters are significantly lower than those of Neck_2, indicating that the two have different performance focuses. Neck_2 focuses on detection accuracy, while Neck_3 focuses on comprehensive target capture and lightweight.

Neck_4: The neck structure of Neck_3 is replaced with the MFHD network. The precision (49.4%), recall rate (39.0%), mAP50 (40.0%), and mAP50:95 (23.9%) comprehensively lead other structures. Although the computational load (19.6) increases, considering the leap in performance, it is within a reasonable range. The number of parameters (1.5 M) is lower than that of Neck_1 and Neck_2 and close to that of Neck_3. This fully illustrates that the MFHD network structure has advantages in multi-scale feature fusion and can integrate feature information of different scales more efficiently. Moreover, while introducing a better feature fusion structure, the network does not excessively sacrifice the efficiency and lightweight advantages of the model, achieving a good balance between performance and efficiency.

### 4.4. Comparison Experiment

#### 4.4.1. Comparative Experiments on Different Neck Networks

The neck network plays a crucial role in feature fusion, enhancement, and transmission. A rationally designed network structure directly affects the model’s capability to extract and utilize small-target features. To verify the advancement of the proposed MFHD neck network, a comparative experiment was constructed. Mainstream feature fusion networks such as PAFPN, GDFPN, HSFPN, and BiFPN were selected. The experimental results are shown in Table 4.

The experimental results indicate that different neck networks have significant differences in influencing model performance:

In terms of detection performance, the proposed MFHD network comprehensively leads PAFPN, GDFPN, HSFPN, and BiFPN in core indicators such as P (49.4%), R (39%), mAP50 (40%), and mAP50:95 (23.9%). This shows that the MFHD network can fuse multi-scale features more efficiently. While accurately identifying targets, it effectively improves the coverage of targets. Whether for small targets or targets of regular scales, it can achieve better detection results.

In terms of model efficiency, the parameter quantity of the MFHD network is 1.5 M, which is much lower than that of other neck networks. The computational load is 19.6 G. Although it is higher than some networks like HSFPN (6.9 G) and BiFPN (7.1 G), considering its substantial lead in detection performance, such an increase in computational cost is acceptable.

Overall, the MFHD architecture demonstrates a notable enhancement in detection accuracy while maintaining model complexity and computational cost. This design highlights its strengths in multi-scale feature integration and structural innovation, confirming both the efficacy and progressiveness of the neck network design.

#### 4.4.2. Comparative Experiments on Different Loss Functions

In the model training process, the selection of the loss function plays a crucial role in determining the model’s performance. The model proposed in this paper adopts InnerCIoU as the loss function during the training phase. To verify its advancement, a comparative experiment was conducted on mainstream localization loss functions in the field of target detection. The CIoU loss function natively used in YOLOv8 was selected as the baseline, and typical loss functions such as DIoU and SIoU were compared and analyzed. The results of the comparative experiment are shown in Table 5.

As can be seen from Table 5, different loss functions have obvious differences in influencing the model’s performance. In terms of the precision (P) indicator, ShapeIoU has the highest value of 52.5%, and InnerCIoU reaches 52.3%, both of which are higher than CIoU (50.43%), DIoU (50.59%), and SIoU (51.58%). This indicates that ShapeIoU and InnerCIoU can more accurately screen out real targets and reduce the false detection rate.

In terms of the recall rate (R), CIoU is 40.7%, and InnerCIoU is 40.1%, which are better than DIoU (40.42%), ShapeIoU (39.42%), and SIoU (39.63%). This shows that CIoU and InnerCIoU have a stronger coverage ability for targets, reducing the missed detection situation.

For the mAP50 indicator, DIoU (41.9%) and InnerCIoU (41.9%) have more advantages compared with other loss functions, reflecting their good performance in target detection under medium IoU thresholds. While in terms of the mAP50:95 indicator, InnerCIoU is the highest. This result indicates that InnerCIoU has better comprehensive detection precision in the entire IoU threshold interval and can maintain a stable and excellent performance in target detection scenarios with different overlap degrees.

Comprehensively, the InnerCIoU loss function used in this paper has achieved good performance in multiple core evaluation indicators. Although it has its own advantages and disadvantages compared with other loss functions in some indicators, it shows good performance in overall performance, verifying its advancement and effectiveness in the training of small-target detection models.

#### 4.4.3. Comparative Experiments on Different NWD Parameters

To verify the influence of the method combining NWD and InnerCIoU introduced in this paper on the small-target detection performance of the RMH-YOLO model, comparative experiments with different α values were conducted to obtain the optimal combination scheme. Five groups of comparative experiments were carried out, respectively, and the experimental results are shown in Table 6.

It can be seen from Table 6 that the weight of α has a relatively large impact on the detection performance of RMH-YOLO. As α increases, the detection precision also increases. Compared with the experimental results of Group G in Table 2, since NWD better balances the differences between samples, when it cooperates with InnerCIoU, it can calculate the optimal solution of the similarity between the predicted box and the real box. Therefore, in this paper, the NWD value α=0.5 is selected to improve the model.

#### 4.4.4. Comparison with Classical Models

To verify the efficiency of the proposed algorithm in small-target detection, mainstream target detection algorithms (ATSS, Cascade-RCNN, Faster-RCNN, TOOD, etc.) were selected. Comparisons were conducted from two dimensions of detection precision and computational efficiency on the VisDrone2019 validation set. Among them, ATSS and GFL are single-stage target detection algorithms, while Cascade-RCNN, Faster-RCNN, and TOOD are two-stage target detection algorithms. The results of comparative experiments for different models are shown in Table 7.

From the perspective of detection precision, the proposed algorithm (ours) shows advantages in core indicators. The precision reaches 53.0%, which is higher than that of single-stage algorithms GFL (47.1%), ATSS (46.4%), and two-stage algorithms Cascade-RCNN (49.6%), Faster-RCNN (48.6%), and TOOD (47.8%). The recall rate is 40.4%, higher than other compared algorithms. The mAP50 is 42.4% and mAP50:95 is 25.7%, surpassing single-stage algorithms GFL (35.4%, 21.0%), ATSS (35.9%, 21.7%), but underperforming the RT-DETR (46.5%, 28.0%) and two-stage algorithms Cascade-RCNN (39.5%, 24.2%), Faster-RCNN (39.2%, 23.5%), TOOD (37.1%, 22.1%), in the comprehensive performance of multi-scale small-target detection.

From the perspective of computational efficiency, the computational load (Flops) of the proposed algorithm is only 16.7 G, and the number of parameters (Params) is 1.3 M. Compared with single-stage algorithms GFL (206.0 G, 32.3 M) and ATSS (110.0 G, 38.9 M), the computational load and number of parameters are greatly reduced. When compared with two-stage algorithms, Cascade-RCNN (236.0 G, 69.3 M), Faster-RCNN (208.0 G, 41.4 M), and TOOD (199.0 G, 32.0 M), RT-DETR(57 G, 19.9 M), the advantages are more significant.

**Figure 10:**
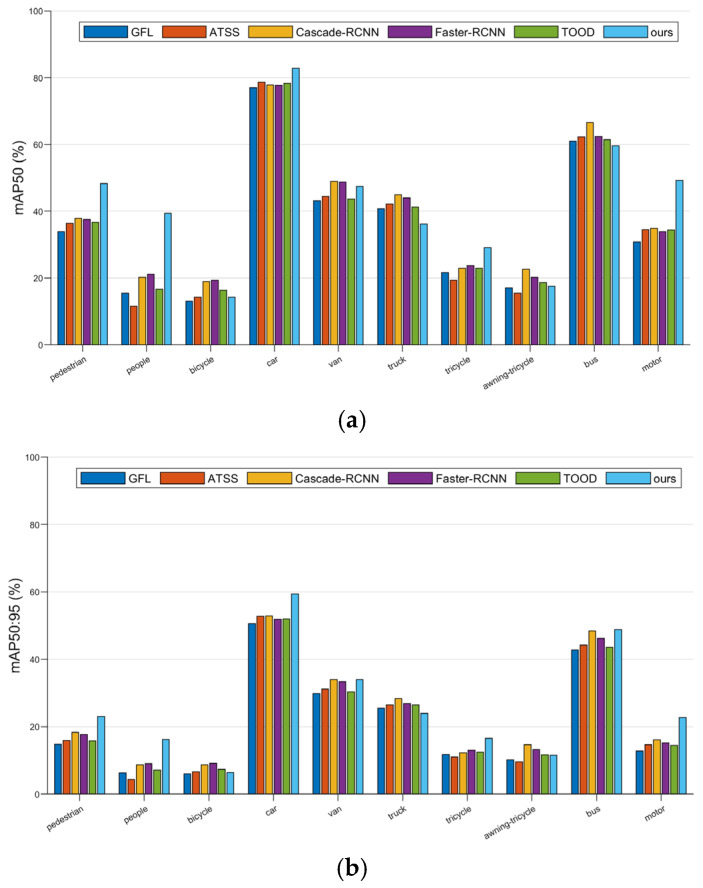
(**a**) Comparison of mAP50 across different object categories among models. (**b**) Comparison of mAP50:95 across different object categories among models.

To evaluate the proposed model’s (ours) small-object detection performance against classical models, analysis of mAP50 (Figure 10a) and mAP50:95 (Figure 10b) shows that in small-object categories (e.g., pedestrian, bicycle, tricycle), our model outperforms classical models for both metrics, and it remains competitive with top classical models in the car category (larger object), validating its effectiveness in small-target detection while handling larger objects well.

In summary, the proposed approach demonstrates clear advantages over existing single-stage and two-stage object detectors in key aspects of small-target recognition, including detection accuracy, computational efficiency, and model compactness, confirming its effectiveness and applicability in real-world UAV-based detection scenarios.

#### 4.4.5. Comparison with YOLO Series Models

**Figure 11:**
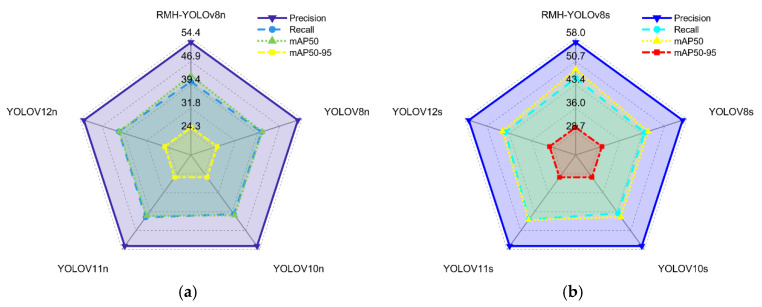
(**a**) Comparison of RMH-YOLOv8n with YOLOn-series models. (**b**) Comparison of RMH-YOLOv8s with YOLOs-series models.

To validate the superiority and effectiveness of the improved algorithm model (RMH-YOLO) proposed in this paper, a series of comparative experiments with YOLO-series models was conducted. The results are presented in Table 8 and Figure 11.

As illustrated in Figure 11a and Table 8, the RMH-YOLOv8n model exhibits comprehensive dominance over comparative YOLOn-series models in all core detection metrics:

- Precision: RMH-YOLOv8n achieves 53.0%, a remarkable improvement over YOLOv8n (44.2%) and YOLOv10n (44.8%), indicating a stronger capability to correctly identify true positive samples.
- Recall: With a recall rate of 40.4%, it surpasses YOLOv8n (33.0%) and YOLOv10n (32.9%), demonstrating superiority in capturing all actual positive samples without omission.
- mAP50: Reaching 42.4%, it far outperforms YOLOv8n (33.2%) and YOLOv10n (33.3%), reflecting robust overall performance at a loose IoU (Intersection over Union) threshold of 0.5.
- mAP50:95: Attaining 25.7%, it leads YOLOv11n (18.8%) and YOLOv12n (18.8%) by a significant margin, highlighting excellent adaptability to different IoU thresholds and high localization accuracy.

Moreover, RMH-YOLOv8n maintains lightweighting with only 1.3 M parameters (vs. YOLOv8n’s 3.0 M). Although its FLOPs (16.7 G) exceed YOLOv8n’s 8.1 G, the substantial accuracy gains justify this trade-off for precision-prioritized scenarios.

For s-scale models (shown in Figure 11b and Table 8), RMH-YOLOv8s showcases even more prominent advantages over comparative YOLOs-series models:

- Precision: Reaching 56.7%, it outperforms YOLOv8s (50.6%) and YOLOv10s (51.0%), emphasizing enhanced ability to filter false positives.
- Recall: With a recall rate of 45.5%, it surpasses YOLOv8s (37.6%) and YOLOv10s (37.9%), ensuring fewer true positives are missed.
- mAP50: Achieving 48.3%, it far exceeds YOLOv8s (38.8%) and YOLOv10s (39.4%), confirming robust performance under IoU = 0.5.
- mAP50:95: At 30.0%, it leads YOLOv8s (23.0%) and YOLOv10s (23.5%), illustrating superior performance across IoU thresholds and high localization precision.

In lightweighting and efficiency, RMH-YOLOv8s reduces parameters from YOLOv8s’ 11.1 M to 5.2 M (benefiting resource-constrained deployments). While its FLOPs rise to 64.8 G (vs. YOLOv8s’ 28.5 G), the ~6.1%/7.9%/9.5% gains in precision, recall, and mAP make this trade-off worthwhile for high-accuracy applications.

Collectively, RMH-YOLOv8n and RMH-YOLOv8s achieve state-of-the-art detection performance while maintaining (or improving) lightweighting, verifying the method’s effectiveness for practical deployment.

#### 4.4.6. Comparison with Other Improved Methods

To further validate the superiority of the proposed RMH-YOLO model, a comparative analysis with other state-of-the-art improved YOLO-based methods is conducted, and the results are presented in Table 9.

From the perspective of detection accuracy, RMH-YOLO achieves a precision (P) of 53.0%, a recall (R) of 40.4%, an mAP50 of 42.4%, and an mAP50:95 of 25.7%. When compared with LE-YOLO (mAP50 = 39.3%, mAP50:95 = 22.7%), ESOD-YOLO (mAP50 = 29.3%, mAP50:95 = 16.6%), RFAG-YOLO (mAP50 = 38.9%, mAP50:95 = 23.1%), TA-YOLO (mAP50 = 40.1%, mAP50:95 = 24.1%), and LSOD-YOLO (mAP50 = 37.0%), RMH-YOLO demonstrates significant advantages in comprehensive detection performance. This indicates that the integrated design of feature enhancement and loss function optimization in RMH-YOLO effectively boosts the model’s capability to recognize small targets in UAV aerial images.

In terms of model size, RMH-YOLO utilizes only 1.3 M parameters, significantly fewer than RFAG-YOLO (5.94 M), TA-YOLO (3.8 M), and LSOD-YOLO (3.8 M). Furthermore, with computational demands of 16.7 G FLOPs, the model achieves greater efficiency compared to LSOD-YOLO (39.0 G), while still delivering strong detection accuracy.

### 4.5. Generalization Experiments

To comprehensively validate the generalization capability of RMH-YOLOv8n and RMH-YOLOv8s, generalization experiments were conducted on the TinyPerson dataset. Similar to VisDrone, TinyPerson focuses on small-object detection in UAV aerial imagery; however, it features even smaller object scales, thereby posing greater challenges to detection models.

**Figure 12:**
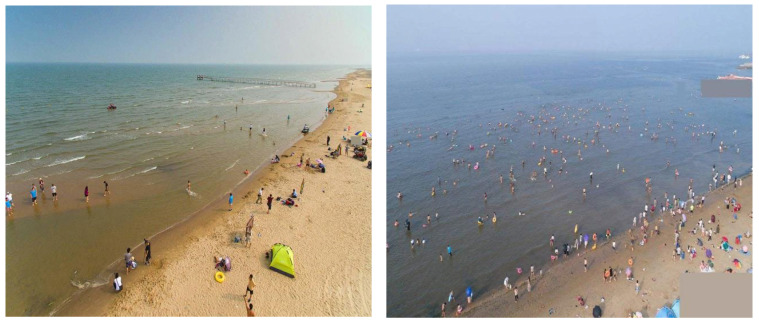
Images of typical scenarios in the TinyPerson dataset.

As depicted in Figure 12, the typical scenario images of the TinyPerson dataset exhibit extremely small target scales (most persons are merely a few pixels under UAV aerial photography), high target density (resulting in severe occlusion and overlap), and complex background interference (where beach and sea water textures easily blur with targets). Collectively, these characteristics amplify the difficulty of small-object detection, rendering TinyPerson a stringent benchmark for assessing model generalization in UAV-based ultra-small object scenarios.

As shown in Table 10, RMH-YOLOv8n outperforms the original YOLOv8n, with improvements in Precision (from 38.9% to 44.4%), Recall (from 25.5% to 33.1%), mAP50 (from 22.6% to 28.5%), and mAP50-95 (from 7.2% to 8.9%). Notably, its Parameters and GFLOPs are reduced to 1.3 and 16.7, respectively, indicating a superior trade-off between detection performance and computational efficiency in small-object generalization tasks. For RMH-YOLOv8s, compared to the original YOLOv8s, it achieves a remarkable improvement in Precision (from 37.7% to 48.3%), accompanied by increases in Recall (from 29.1% to 33.7%), mAP50 (from 25.3% to 30.5%), and mAP50–95 (from 8.1% to 9.8%). Although its Parameters and FLOPs increase, the substantial accuracy gains confirm the efficacy of RMH modifications in generalizing to datasets like TinyPerson with extremely small object scales.

In summary, the experimental results on TinyPerson, coupled with the analysis of its typical scenario images, fully demonstrate that RMH-YOLOv8n and RMH-YOLOv8s exhibit strong generalization capabilities in UAV-based small-object detection tasks, particularly in scenarios with ultra-small object scales.

## 5. Visualization Experiment

### 5.1. Detection Results Visualization

**Figure 13:**
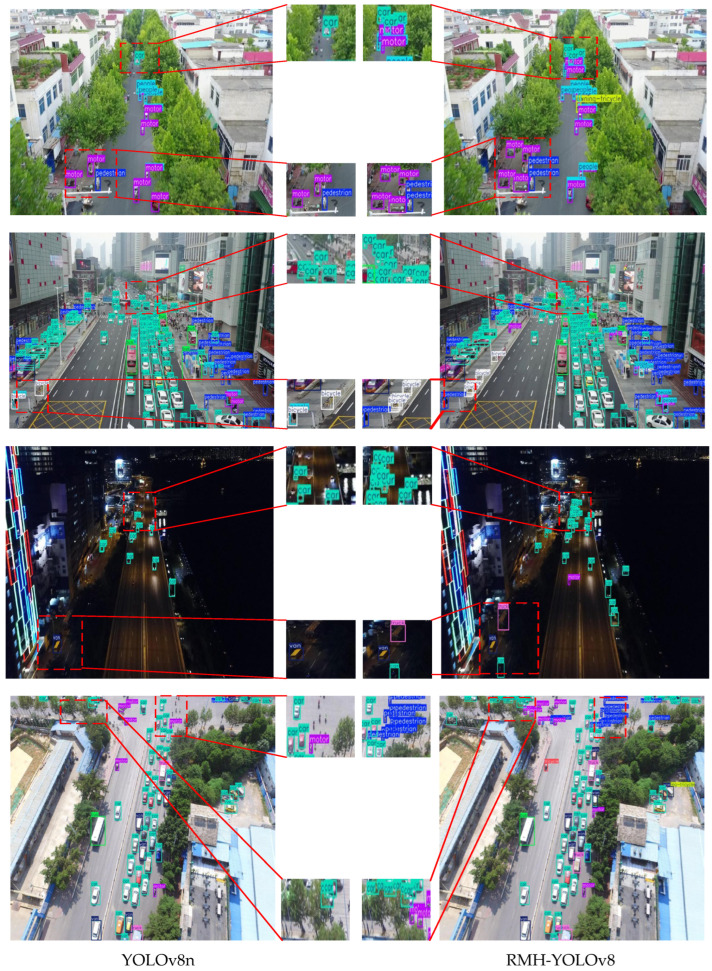
Comparison of detection results in various scenarios.

To comprehensively evaluate the detection performance of RMH-YOLOv8 under diverse scenarios, four representative scenarios were selected from the VisDrone-2019 dataset: complex background scenes, long-range dense target scenes, low-light target scenes, and strong light target scenes. The detection results of YOLOv8n and RMH-YOLOv8 in these four typical scenarios are illustrated in Figure 13. In each group of comparison images, the left side presents the detection results of the YOLOv8n model, while the right side shows the detection results of the RMH-YOLOv8 model. Red rectangular boxes are utilized to highlight the differences in detection results between the two models.

From the detection results in Figure 13, it can be observed that for the small-target detection task against complex backgrounds, due to the relatively low pixel proportion of small targets in the images, the YOLOv8n baseline model exhibits a significant missed detection phenomenon. In contrast, the proposed RMH-YOLOv8 model can reliably accomplish small-target detection tasks in complex backgrounds. Even when targets are partially occluded, it can still achieve effective recognition and localization of the targets. In long-range target detection scenarios, affected by the dramatic changes in target scale, the missed detection problem of the YOLOv8n model is further aggravated. However, the RMH-YOLOv8 model significantly improves upon the baseline model, effectively reducing the missed detection rate in long-range small-target detection tasks.

Moreover, in low-light night scenes and strong light scenes, the RMH-YOLOv8 model demonstrates stronger environmental adaptability and detection robustness compared to the YOLOv8n baseline model. Nevertheless, it should be noted that when there is a problem of feature blurring caused by the slow movement of small targets in night scenes, the detection precision of the RMH-YOLOv8 model still has room for improvement. This issue also provides a direction for subsequent research optimization.

### 5.2. Heatmap Visualization

**Figure 14:**
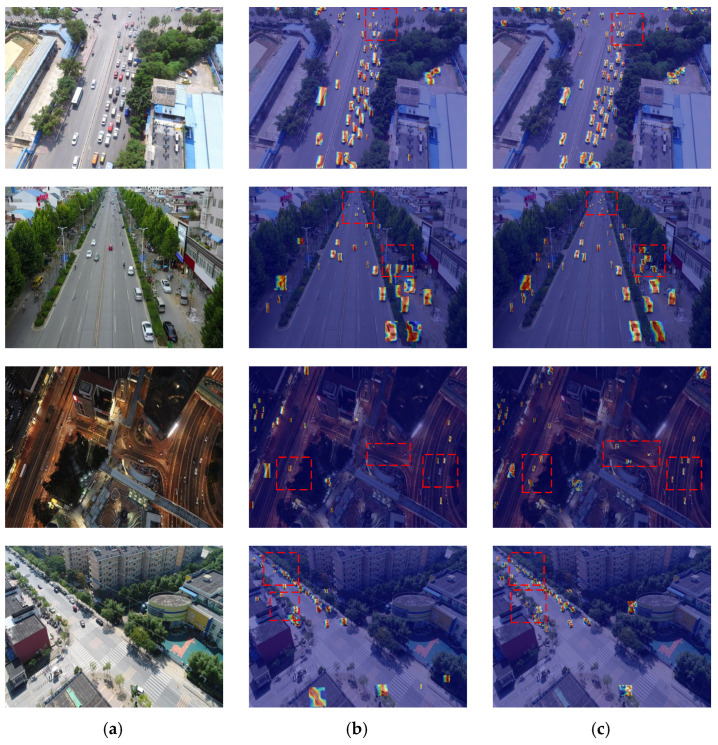
Heatmap comparison across different models. (**a**) Original pictiure; (**b**) YOLOv8n; (**c**) ours.

To intuitively illustrate the superior small-target detection capability of our proposed model, heatmap visualization is employed to depict the receptive fields and attention regions of the models. As shown in Figure 14, which presents heatmaps derived from the backbones of different models on images from the VisDrone2019 dataset across various scenarios, the first column displays the original images, the second column shows the heatmaps of the baseline model (YOLOv8n) backbone, and the third column presents the heatmaps of our model’s backbone. In heatmap visualization, deeper colors signify that the model pays more attention to the corresponding regions.

In the dense small-target scenario (first row), the heatmap of the YOLOv8n backbone exhibits relatively scattered and less intense attention on small targets, with some small targets even lacking a distinct color indication. In contrast, our model’s backbone heatmap shows concentrated and deeper-colored regions precisely corresponding to the dense small targets. This indicates that our model can effectively focus on and capture features of small targets in dense distributions, which is crucial for detecting numerous small targets simultaneously.

For the scenario with different-scale targets in complex backgrounds (second row), the YOLOv8n backbone heatmap has difficulty in uniformly and accurately focusing on targets of various scales, especially small ones, which are often overshadowed by the complex background in terms of attention. However, our model’s backbone heatmap clearly highlights targets of different scales, including small ones, with prominent coloration. This demonstrates that our model can better distinguish small targets from complex backgrounds and allocate appropriate attention to them. In the nighttime scenario with fast-moving small targets (third row), the YOLOv8n backbone heatmap fails to stably focus on the fast-moving small targets, resulting in blurred or weak attention regions. Our model’s backbone heatmap, on the other hand, maintains clear and deep attention on these small targets despite their movement, showing that our model can effectively track and capture the features of fast-moving small targets in low-light conditions. Regarding the scenario of small-target detection under strong exposure (fourth row), the YOLOv8n backbone heatmap is disturbed by the strong exposure, leading to inaccurate or diffused attention on small targets. Meanwhile, our model’s backbone heatmap can still accurately focus on small targets, with distinct and deep-colored attention regions. This reflects that our model is robust to strong exposure and can reliably detect small targets in such challenging lighting conditions.

Overall, across all these scenarios, our model’s backbone demonstrates more accurate, concentrated, and robust attention on small targets compared to the YOLOv8n backbone. This superior attention mechanism enables our model to effectively capture the features of small targets in various complex environments, thereby explaining the effectiveness of our model in small-target detection.

## 6. Conclusions

Small-target detection in UAV aerial imagery represents a critical yet challenging task for aerial monitoring and remote sensing applications. The task is complicated by several inherent characteristics: targets with minimal pixel representation due to large field-of-view and varying flight altitudes; substantial scale variations arising from dynamic changes in perspective and altitude; complex backgrounds that readily obscure small targets; and significant illumination variations that degrade imaging quality and feature discriminability.

While previous research has advanced UAV-based small-target detection, existing approaches exhibit notable limitations. Traditional methods fail to fully exploit multi-scale feature information, and conventional feature fusion architectures often result in the loss of fine-grained spatial details from shallow feature layers—information that is critical for accurately localizing small targets in aerial imagery. These limitations lead to increased false detections and missed detections, particularly for small objects with weak visual signatures.

To address these challenges, this study proposes RMH-YOLO, a refined multi-scale architecture optimized for small-target detection in UAV aerial imagery. The model incorporates four targeted architectural improvements. First, a Refined Feature Module (RFM) integrated into the backbone network effectively enhances feature extraction from low-resolution imagery, strengthening the representation of weak small-target features through dual-attention mechanisms. Second, a Multi-scale Focus-and-Diffuse (MFFD) network replaces the original PAFPN structure, employing a focus-diffuse transmission pathway to preserve fine-grained spatial details from high-resolution layers while propagating them to semantic-rich features, thereby reducing information loss during multi-scale feature fusion. Third, an efficient detection head (CS-Head) utilizing parameter-sharing convolution enables efficient processing on embedded platforms while eliminating redundant parameters and maintaining detection capability for small targets. Fourth, an optimized loss function combining InnerCIoU with Normalized Wasserstein Distance (NWD) improves bounding box regression for small targets, enabling more effective parameter updates and enhanced localization accuracy.

Comprehensive experimental validation on the VisDrone2019 dataset demonstrates the effectiveness of RMH-YOLO. Compared with the baseline YOLOv8n model, RMH-YOLO achieves substantial improvements: precision and recall increase by 8.8% and 7.4%, respectively, while mAP50 and mAP50:95 improve by 9.2% and 6.4%. These performance gains are achieved while maintaining computational efficiency with only 1.3 M parameters and 16.7 G FLOPs, making the model suitable for deployment on resource-constrained UAV platforms.

In summary, RMH-YOLO provides an effective solution that balances detection accuracy with computational efficiency for small-target detection in UAV aerial imagery. The proposed architectural innovations—particularly the focus-diffuse feature fusion strategy and dual-attention enhancement mechanisms—demonstrate significant value for improving small-object recognition in aerial monitoring applications. These results confirm the model’s suitability for deployment on embedded UAV platforms, demonstrating its broad application potential in diverse UAV aerial monitoring scenarios.

## Figures and Tables

**Table 1:** Experimental Conditions of Overall Module Ablation Tests.

Group	Baseline	RFAT	MFHD	CSLD	InnerCIoU	NWD
A	√					
B	√	√				
C	√		√			
D	√			√		
E	√	√	√			
F	√	√	√	√		
G	√	√	√	√	√	
H	√	√	√	√	√	√

**Table 2:** Results of Overall Module Ablation Tests.

Group	P/%	R/%	mAP50/%	mAP50:95/%	Parameters/M	FLOPs/G	FPS
A	44.2	33.0	33.2	19.3	3.0	8.1	134.8
B	46.7	35.1	35.7	21.1	3.4	10.4	112.9
C	49.4	39.0	40.0	23.9	1.5	19.6	84.2
D	43.9	32.7	32.7	19.0	2.4	6.5	151.8
E	51.6	41.0	42.0	25.2	1.8	21.6	91.5
F	50.4	40.7	41.6	25.1	1.3	16.7	76.6
G	52.3	40.1	41.9	25.5	1.3	16.7	76.5
H	**53.0 (+8.8)**	**40.4 (+7.4)**	**42.4 (+9.4)**	**25.7 (+6.4)**	**1.3 (−1.7)**	**16.7 (+8.6)**	76.5

**Table 3:** MFHD Ablation Tests.

Group	P/%	R/%	mAP50/%	mAP50:95/%	Parameters/M	FLOPs/G	FPS
Neck_1	44.2	33.0	33.2	19.3	3.0	8.1	134.8
Neck_2	47.7	35.5	36.7	21.8	2.9	12.2	124.2
Neck_3	44.7	35.9	35.8	21.2	1.0	10.4	127.4
Neck_4	49.4	39.0	40.0	23.9	1.5	19.6	76.6

**Table 4:** Comparative Experiment on Different Neck Networks.

Neck	P/%	R/%	mAP50/%	mAP50:95/%	Parameters/M	FLOPs/G	FPS
PAFPN (baseline)	44.2	33	33.2	19.3	3.0	8.1	134.8
GDFPN [26]	44.7	33.2	33.2	19	3.3	8.3	131.9
HSFPN [27]	42.7	31.4	31.2	18	1.9	6.9	109.9
BiFPN [28]	44.7	32.9	33.4	19.5	2.0	7.1	102.8
MFHD (ours)	49.4	39	40.0	23.9	1.5	19.6	76.5

**Table 5:** Comparative Experiment on Different Loss Functions.

Loss	P/%	R/%	mAP50/%	mAP50:95/%
CIoU	50.4	40.7	41.6	25.1
DIoU	50.6	40.4	41.9	25.3
ShapeIoU	52.5	39.4	41.6	25.2
SIoU	51.6	39.6	41.2	25.0
InnerCIoU (ours)	52.3	40.1	41.9	25.5

**Table 6:** Comparative Experiment with Different α Values.

α	P/%	R/%	mAP50/%	mAP50:95/%
0.1	53.2	40.6	42.3	25.2
0.3	52.3	40.0	42.2	25.4
0.5	53.6	40.1	42.2	25.5
0.7	52.0	40.5	41.9	25.3
0.9	51.3	40.5	41.6	25.1

**Table 7:** Results of Comparative Experiments with Different Models.

Model	P/%	R/%	mAP50/%	mAP50:95/%	Params/M	Flops/G	FPS
GFL [29]	47.1	36.2	35.4	21.0	32.3	206.0	15.2
ATSS [30]	46.4	37.8	35.9	21.7	38.9	110.0	35.1
Cascade-RCNN [31]	49.6	37.3	39.5	24.2	69.3	236.0	30.7
Faster-RCNN [32]	48.6	37.2	39.2	23.5	41.4	208.0	17.4
TOOD [33]	47.8	37.6	37.1	22.1	32.0	199.0	18.6
RT-detr [34]	60.4	45.1	46.5	28.0	19.9	57	69.8
MFHD (ours)	53.0	40.4	42.4	25.7	1.3	16.7	76.5

**Table 8:** Comparative experiments of YOLO-series algorithms.

Model	P/%	R/%	mAP50/%	mAP50:95/%	Parameters/M	FLOPs/G
RMH-YOLOv8n	53.0	40.4	42.4	25.7	1.3	16.7
YOLOV8n	44.2	33.0	33.2	19.3	3.0	8.10
YOLOV10n	44.8	32.9	33.3	19.1	2.3	6.50
YOLOV11n	42.7	32.7	32.2	18.8	2.6	6.30
YOLOV12n	42.8	32.4	32.3	18.8	2.6	6.30
RMH-YOLOv8s	56.7	45.5	48.3	30.0	5.2	64.8
YOLOV8s	50.6	37.6	38.8	23.0	11.1	28.50
YOLOV10s	51.0	37.9	39.4	23.5	7.2	21.4
YOLOV11s	48.7	38.9	39.1	23.4	9.4	21.3
YOLOV12s	49.5	37.5	38.5	23.1	9.2	21.2

**Table 9:** Comparison with Other Improved Methods.

Model	P/%	R/%	mAP50/%	mAP50:95/%	Parameters/M	FLOPs/G
RFAG-YOLO [35]	49.6	37.8	38.9	23.1	5.94	15.7
LE-YOLO [36]	-	-	39.3	22.7	2.1	13.1
ESOD-YOLO [37]	-	-	29.3	16.6	4.47	14.3
TA-YOLO [38]	50.2	37.3	40.1	24.1	3.8	-
LSOD-YOLO [39]	48.4	38.2	37	-	3.8	33.9
RMH-YOLOv8n	53.0	40.4	42.4	25.7	1.3	16.7
RMH-YOLOv8s	56.7	45.5	48.3	30.0	5.2	64.8

**Table 10:** Experimental results for TinyPerson.

Model	P/%	R/%	mAP50/%	mAP50:95/%	Parameters/M	FLOPs/G
YOLOv8n	38.9%	25.5%	22.6%	7.2%	3.0	8.1
RMH-YOLOv8n	44.4%	33.1%	28.5%	8.9%	1.3	16.7
YOLOv8s	37.7%	29.1%	25.3%	8.1%	11.1	28.4
RMH-YOLOv8s	48.3%	33.7%	30.5%	9.8%	5.2	64.7

## Data Availability

The original contributions presented in the study are included in the article. Further inquiries can be directed to the corresponding author.
